# HIV-1 DIS stem loop forms an obligatory bent kissing intermediate in the dimerization pathway

**DOI:** 10.1093/nar/gku332

**Published:** 2014-05-09

**Authors:** Hansini Mundigala, Jonathan B. Michaux, Andrew L. Feig, Eric Ennifar, David Rueda

**Affiliations:** 1 Department of Chemistry, Wayne State University, Detroit, MI 48236, USA; 2 Architecture et Réactivité de l'ARN, Université de Strasbourg, Institut de Biologie Moléculaire et Cellulaire du CNRS, F-67084 Strasbourg, France; 3 Department of Medicine, Section of Virology, Imperial College, London W12 0NN, UK; 4 Single Molecule Imaging Group, MRC Clinical Sciences Center, Imperial College, London W12 0NN, UK

## Abstract

The HIV-1 dimerization initiation sequence (DIS) is a conserved palindrome in the apical loop of a conserved hairpin motif in the 5′-untranslated region of its RNA genome. DIS hairpin plays an important role in genome dimerization by forming a ‘kissing complex’ between two complementary hairpins. Understanding the kinetics of this interaction is key to exploiting DIS as a possible human immunodeficiency virus (HIV) drug target. Here, we present a single-molecule Förster resonance energy transfer (smFRET) study of the dimerization reaction kinetics. Our data show the real-time formation and dissociation dynamics of individual kissing complexes, as well as the formation of the mature extended duplex complex that is ultimately required for virion packaging. Interestingly, the single-molecule trajectories reveal the presence of a previously unobserved bent intermediate required for extended duplex formation. The universally conserved A272 is essential for the formation of this intermediate, which is stabilized by Mg^2+^, but not by K^+^ cations. We propose a 3D model of a possible bent intermediate and a minimal dimerization pathway consisting of three steps with two obligatory intermediates (kissing complex and bent intermediate) and driven by Mg^2+^ ions.

## INTRODUCTION

Human immunodeficiency virus (HIV), a retrovirus, attacks the human immune system, which can result in acquired immune deficiency syndrome (AIDS) (1–3). HIV is the leading cause of death in Africa and the fourth leading cause of death worldwide (4,5). Current therapies against HIV target mainly two viral enzymes: reverse transcriptase (6) and protease (7). Due to the rapid evolution of strains resistant to enzymatic inhibitors, new targets must be identified.

HIV contains two similar copies of its genomic RNA (gRNA), which share numerous intermolecular interactions (8). The most prominent one is the dimer linkage structure (DLS) in the 5′-untranslated region (UTR) of the viral genome (9,10), wshich has been shown to control translation, RNA packaging and recombination during proviral DNA synthesis. It has also been shown that multiple structural transitions in the 5′ UTR can regulate gRNA packaging (11,12).

Within the DLS, a highly conserved, nine-nucleotide apical loop, including a six-nucleotide, palindromic, dimerization initiation sequence (DIS), has been shown to be important in the dimerization process. The palindromic sequence is flanked by three conserved purines (A272, A/G273 and A280) that are essential for the dimer stability (13–15). The interaction between the DLS of the two gRNA is initiated by formation of a kissing loop at the DIS (16,17). Mutations or alterations of the DIS prevents RNA dimerization and severely reduces the viral infectivity (18–21). It has been shown that the 35 nucleotide (nt) DLS with the lower stem bulge is required for the two-step dimerization in presence of NCp7 whereas the 23 nt construct can achieve the two-step dimerization in absence of this protein (22–24). Experiments on synthetic RNA fragments have shown that the initial kissing-loop dimer is subsequently stabilized by extension of intermolecular Watson–Crick base pairs as an extended duplex (ED) (16,25). This kissing loop to ED isomerization is strongly facilitated *in vitro* by incubation at high temperature (55°C), or at physiological temperature by the nucleocapsid protein (NC), a small, basic protein with two zinc-finger domains (22,23,25–29). Structural insights of the DIS kissing loop and ED forms have been provided by X-ray crystallography (30–32) and nuclear magnetic resonance (NMR) (33–35). This structural work has led to the discovery of high-affinity ligands of both the kissing loop and ED forms (36–39), thus highlighting the DIS as a potentially interesting new viral RNA target. However, despite these progresses, little is known about the exact mechanism of isomerization.

To further investigate the isomerization mechanism, we have employed single-molecule Förster resonance energy transfer (smFRET) to dissect, in real-time, the *in vitro* dimerization reaction of 23 nt RNAs containing HIV-1 DIS in absence of NC. The data show that the dimerization pathway proceeds via three steps with two obligatory intermediate conformations: the kissing complex (KC) and a bent intermediate. Progression along the pathway largely requires the presence of Mg^2+^ ions, which stabilize the bent intermediate. Conversely, monovalent ions, such as K^+^, impede ED formation by stabilizing the KC over the bent intermediate. Mutations of the highly conserved purines in the loop indicate that the KC stability and formation of the bent intermediate require a Mg^2+^-dependent conformational change in the purines.

## MATERIALS AND METHODS

### RNA purification and labeling

**Figure 1.**
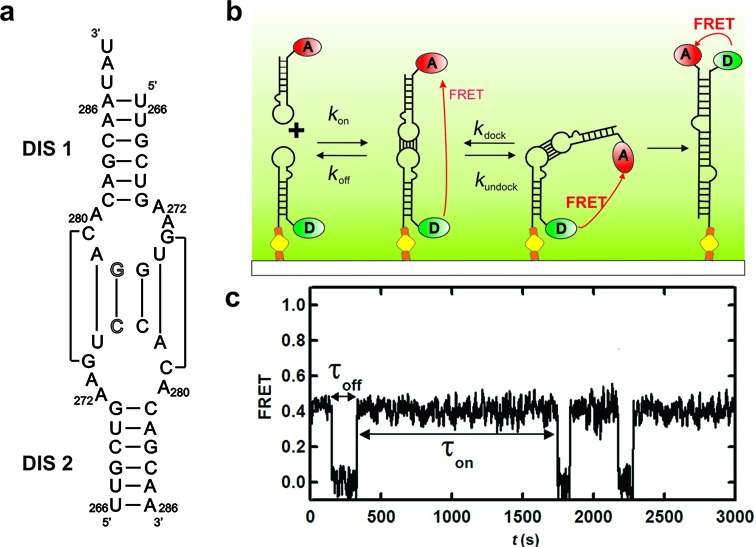
smFRET detection of the bimolecular HIV-1 RNA dimerization. (**a**) Secondary structure of the RNA strands used in this study with the fluorophores. A single base-flip mutation in the center base pair of the loop is used to prevent homodimerization and promote dimerization of Cy3–Cy5 labeled hairpins. (**b**) Schematic diagram of the single molecule experiments. A Cy3 labeled DIS-1 hairpin is surface-immobilized onto the quartz slide via a biotin-streptavidin bridge. Cy5 labeled DIS-2 hairpin is introduced into the slide and allowed to interact freely with the immobilized DIS-1 under near-physiological conditions (20 mM TRIS pH 7.4, 5 mM Mg^2+^ and 150 mM KCl). The fluorophores are excited via a prism-based total internal reflection microscope. Fluorescence is collected through the objective, separated with appropriate dichroic mirrors and monitored with a CCD camera. The dimerization pathway consists of three possible FRET states: free DIS-1 (0 FRET), the kissing complex (0.4 FRET) and the extended duplex (1.0 FRET). (**c**) Resulting FRET time trajectory shows the formation (FRET ∼ 0.4) and dissociation (FRET ∼ 0) of the kissing-loop complex. Dwell times in each state (τ_on_ and τ_off_) are used to build the dwell time distribution (see Figure 2).

RNA samples were purchased from the Keck Foundation Resource Laboratory at the Yale University School of Medicine with a 5′-Cy3 and a 3′-biotin (DIS-1, Figure 1a) or with a 3′-C7 amino linker (DIS-2, Figure 1a). An intermolecular GC base pair in the loop was flipped to prevent homodimer formation while maintaining the KC stability (Figure 1a and Supplementary Materials). RNAs were deprotected, purified and labeled as previously described (40,41). Briefly, deprotected RNAs were purified by denaturing gel electrophoresis (20% wt/vol polyacrylamide and 8 M urea) and diffusion elution against elution buffer (0.5 M NH_4_OAc and 0.1 mM EDTA) overnight at 4°C, followed by chloroform extraction, ethanol precipitation and C8 reverse-phase High Performance Liquid Chromatography (HPLC). The C7 amino linker in DIS2 was labeled with Cy5 (GE Healthcare) in labeling buffer (100 mM Na_2_CO_3_, pH 8.5) overnight at room temperature. The labeled RNA was further purified by ethanol precipitation and reverse-phase HPLC using a linear gradient of acetonitrile in triethylammonium acetate as mobile phase, as described (40). RNA concentrations were determined by UV-Vis absorbance at 260 nm and absorbance at wavelengths of 550 nm and 650 nm were obtained to quantify Cy3 and Cy5 dye incorporation.

### Single-molecule FRET

Single molecule experiments were performed as previously described (42,43). Briefly, RNA strands DIS-1 and DIS-2 were diluted to 25 pM and 30 nM, respectively, in standard buffer (20 mM Tris-HCl pH 7.5, 150 mM KCl and 5 mM MgCl_2_ in saturating trolox). DIS-1 was heated at 90°C for 45 s before flash cooling on ice to prevent homodimerization. DIS-1 RNA was surface-immobilized onto streptavidin-coated quartz slide via a biotin-streptavidin bridge (Figure 1b) to generate a surface density of ∼0.1 molecules/μm^2^. DIS-2 was manually flowed into the slide microchannel in standard buffer with an oxygen-scavenging system (5 mM 3,4-dihydroxybenzoic acid and 0.1 μM protocatechaute 3,4-dioxygenase) to minimize photobleaching. Cy3 was excited in a home-built total internal reflection microscope with a laser (532 nm, 3 mW, Spectra-Physics, Excelsior). Donor and acceptor emissions were separated using appropriate dichroic mirrors (610DCXR, Chroma) and detected as two side-by-side images on a back-illuminated electron-multiplied CCD camera (I-Xon, Andor). Measurements were obtained under variable [Mg^2+^] and [K^+^] (0.001–20 mM and 0–1 M, respectively) at room temperature. Dynamic FRET traces were rarely observed in the time duration of experiments (15–20 min) with a time resolution of 33 ms. In order to maximize the time duration of experiments, 1000 ms time resolution was used and time trajectories were recorded with one frame per second time resolution for up to 45–60 min (Figure 1c). Trajectories were time-binned to construct FRET histograms, and dwell times calculated for each dimerization event to determine the rate constants, as described (45). FRET cutoff values of 0.25 and 0.75 were used to distinguish between monomer and KC and ED. Dwell time histograms were fit to either single or double exponentials to determine *k*_on_ and *k*_off_. Metal ion (Mg^2+^ and K^+^) titrations were fit to the modified Langmuir Equation (1) to obtain the binding constants (*K*_1/2_):

(1) \begin{equation*} f(x) = f_0 + (f_{\max } - f_0 )\frac{x}{{K_{1/2} + x}}, \end{equation*}

where *f_0_* and *f*_max_ are the initial and saturating populations, respectively, and *x* is the concentration of Mg^2+^ or K^+^ ions.

### Model building

Modeling was carried out starting from the HIV TAR RNA/SELEX RNA kissing-loop complex (PDB ID 2RN1) (44) using the program Coot (46). Residues of the stem and the loop were mutated to match the DIS sequence and each stem length was extended to seven base pairs. Stem/loop junctions were opened in order to insert flanking adenines 5′ and 3′ of the loop, without changing the angle between each stem. Adenines 280 were modeled stacked inside the helix whereas adenines 272 and 273 were placed outside the helix and stacked on each other, similarly to their respective conformation observed in the HIV DIS kissing-loop complex crystal structure (PDB ID 1XPF) (32). After model building, several cycles of geometrical regularization were carried out with Coot.

## RESULTS

### smFRET reveals that kissing complex formation and dissociation is an extraordinarily slow process

**Figure 2.**
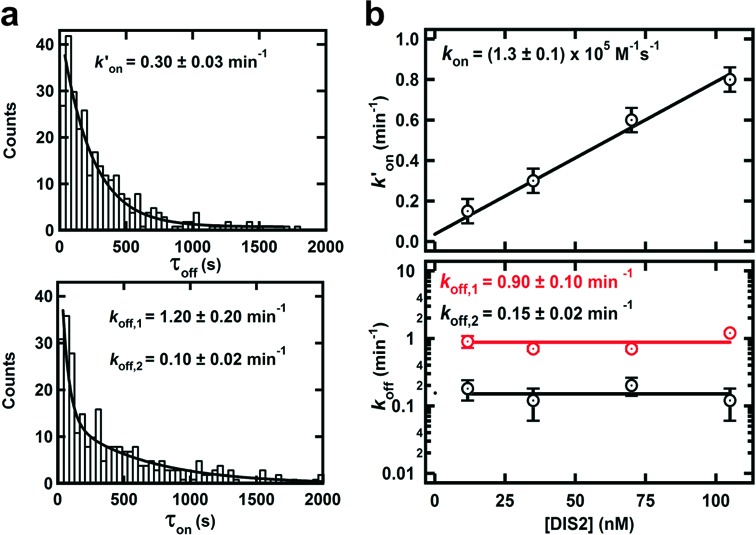
Single molecule kinetic data analysis. (**a**) Dwell time distributions in the monomer DIS1 form (top, τ_off_, FRET ∼ 0) for >100 single-molecule trajectories. Distributions are fit to a single exponential decay to obtain the pseudo-first-order binding rate constant *k*′_on_. Dwell time distributions in the kissing complex (bottom, τ_on_, FRET ∼ 0.4) for >100 single-molecule trajectories. Distributions are fit to double exponential decays to obtain the dissociation rate constants, *k*_off,1_ and *k*_off,2_. (**b**) Dependence of the pseudo-first-order binding rate constant (*k*′_on_) as a function of DIS2 concentration (top). The linear dependence confirms first-order kinetics relative to DIS2, and the slope yields the second-order rate constant (*k*_on_). Dependence of the dissociation rate constant *k*_off_ as a function of DIS2 concentration (bottom). The lack of dependence of *k*_off_ on DIS2 concentration confirms that dissociation is a unimolecular process. These experiments were performed at near-physiological conditions (20 mM TRIS pH 7.4, 5 mM Mg^2+^ and 150 mM KCl).

To dissect the *in vitro* dimerization reaction of DIS RNA (Figure 1a), we have used a smFRET assay previously developed to monitor the KC formation of RNA hairpins in general (45,47,48). The FRET donor-labeled hairpin (DIS1) is surface-immobilized via a biotin-streptavidin bridge while the FRET acceptor-labeled hairpin (DIS2) diffuses in standard buffer under near-physiological conditions (Figure 1b). A typical single molecule FRET time trajectory (Figure 1c) reveals the presence of random excursions between two distinct states at ∼0.0 and ∼0.4 FRET, corresponding to DIS1 monomer and the DIS1:DIS2 KC, respectively. Using the latter FRET value and typical approximations (κ^2^ = 2/3 and *R*_0_ = 60 Å for Cy3–Cy5) (49), we estimate the distance between the two fluorophores to be ∼64 Å, in excellent agreement with the KC crystal structure (31). The time trajectories reveal that the hairpins can undergo many cycles of association and dissociation without progressing to ED formation and that the KC can be very long-lived (>10 min). A dwell-time analysis in the monomer (τ_off_) and KC (τ_on_) states yields the pseudo-first-order association (*k*′_on_) and dissociation (*k*_off_) rate constants, respectively (Figure 2a). The resulting histograms confirm the slow association and dissociation kinetics, as well as heterogeneous dissociation kinetics (double exponential), indicating that two KC populations with different stabilities (i.e. loop–loop interactions) may be formed. As expected for a binary reaction, *k*_off_ is independent of the RNA concentration, while *k*′_on_ increases linearly with [DIS2] (Figure 2b). A linear fit to the latter yields the second-order binding rate constant *k*_on_ = 10^5^ M^−1^s^−1^. This extremely slow formation rate constant may be the result of structural rearrangement of the hairpin prior to forming the KC, which makes most diffusion-controlled collisions between the two hairpins unsuccessful. To further investigate the origin of the two KC populations, we conducted metal ion titrations.

### Mg^2+^ ions stabilize the kissing complex

**Figure 3.**
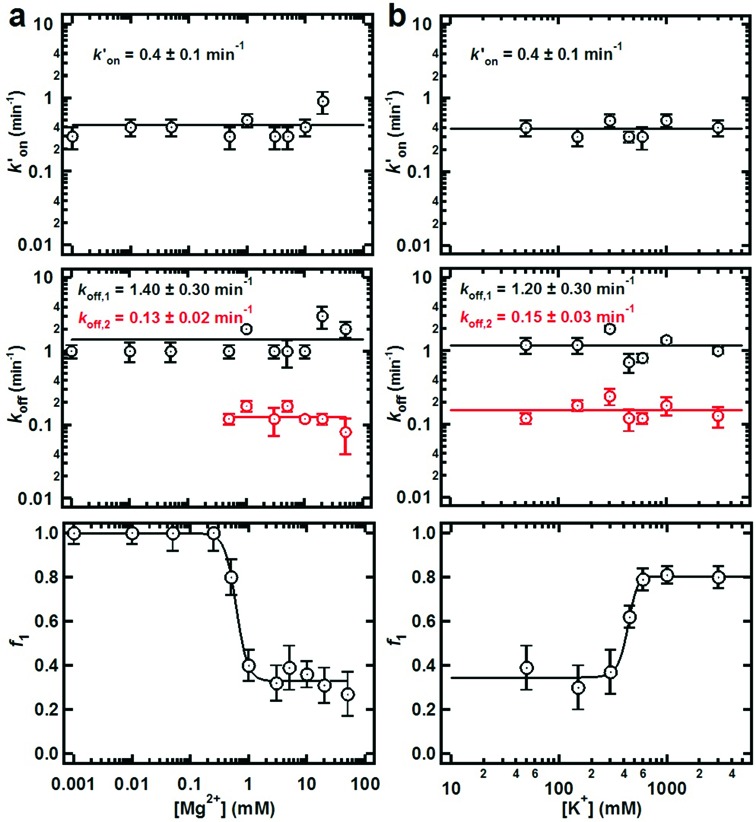
Effect of ionic strength on the dimerization dynamics. (**a**) Top graph: dependence of the pseudo-first-order binding rate constant *k*′_on_ as a function of [Mg^2+^]. *k*′_on_ is independent of [Mg^2+^] indicating that Mg^2+^ ions are not essential in the rate limiting step of KC formation. Middle graph: dependence of the first-order dissociation rate constants *k*_off,1_ and *k*_off,2_ as a function of [Mg^2+^]. The fast dissociation rate constant (*k*_off,1_) in low [Mg^2+^] indicates the presence of a metastable KC, possibly missing essential Mg^2+^ ions for stability. A slow dissociation rate constant (*k*_off,2_) appears at high [Mg^2+^] indicating the presence of a second, stable KC with incorporated Mg^2+^ ions that stabilize the kissing interaction. Bottom graph: fraction of fast dissociation rate constant (*k*_off,1_) decreases with increasing [Mg^2+^]. This result is in agreement with the idea that the slow dissociating KC population arises as a result of Mg^2+^ binding to the KC. These experiments were performed at 20 mM TRIS pH 7.4 and 150 mM KCl. (**b**) Top graph: dependence of the pseudo-first-order binding rate constant *k*′_on_ as a function of [K^+^]. Middle graph: dependence of the first-order dissociation rate constants *k*_off,1_ and *k*_off,2_ as a function of [K^+^]. Bottom graph: fraction of fast dissociation rate constant (*k*_off,1_) increases with increasing [K^+^]. This result indicates that it requires ∼500 mM [K^+^] to compete with the bound Mg^2+^ ions. These experiments were performed at 20 mM TRIS pH 7.4 and 5 mM Mg^2+^.

To test whether the observed heterogeneity results from different metal ion interactions, we measured *k*′_on_ and *k*_off_ as a function of [Mg^2+^] and [K^+^] (Figure 3). The observed *k*′_on_ is independent of both [Mg^2+^] and [K^+^] (Figure 3, top). Interestingly, the long-lived KC population with a slow dissociation constant (*k*_off,2_) is only observed at [Mg^2+^] ≥ 0.5 mM, whereas the short-lived KC population (*k*_off,1_) is observed across the entire [Mg^2+^] range (Figure 3a, middle). The data show that the magnitude of both *k*_off,1_ and *k*_off,2_ remains invariant with [Mg^2+^], but the fraction of the short-lived KC population (*f*_1_) decreases sharply near 0.5 mM [Mg^2+^] in favor of the long-lived population (Figure 3a, bottom). This result implies that the slow population arises from binding of a specific magnesium ion to the hairpin with a dissociation constant of 0.5 mM and stabilizing the KC by ∼2 kcal mol^−1^. This result is in agreement with the presence of a Mg^2+^ ion in the crystal structure of the DIS KC and with prior biochemical and biophysical bulk experiments (50,51).

To test for the specificity of this magnesium ion interaction, we titrated potassium ions in a background of 5 mM Mg^2+^. The data show that, above 300 mM, K^+^ ions can partially recover the fast dissociating population (*f*_1_) indicating that monovalent ions can compete against the divalent ion for the binding site. However, the large amount of K^+^ ions required for this competition supports the notion that a tightly and specifically bound Mg^2+^ ion is primarily responsible for the observed high stability of the KC.

### smFRET reveals an obligatory bent intermediate

**Figure 4.**
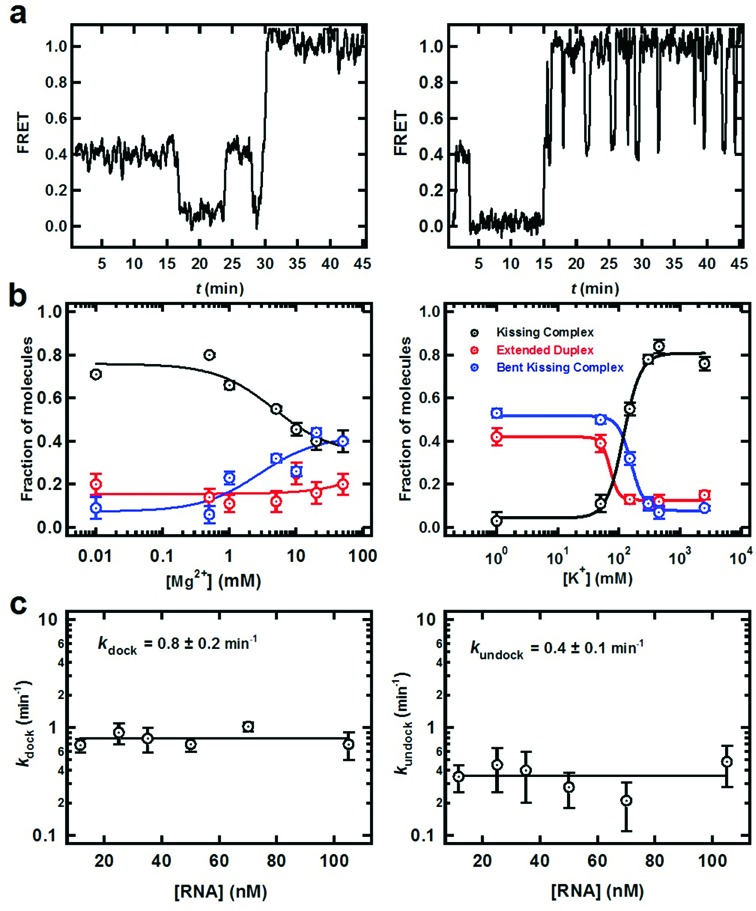
Presence of an obligatory bent intermediate on the dimerization pathway. At all experimental conditions, three different types of single molecule trajectories were observed: (i) trajectories exhibiting dynamics between monomer RNA (FRET 0.0) and KC (FRET 0.4), (ii) trajectories exhibiting the formation of the extended duplex (static FRET 1.0), from the KC form, and (iii) trajectories exhibiting the formation of a dynamic intermediate (Dynamic FRET 1.0). Representative FRET time trajectory showing (**a**) the static FRET 1.0 indicating the formation of the stable extended duplex (left) and dynamic FRET 1.0 state (intermediate dimer, right). (**b**) Variation of the fraction of molecules exhibiting the intermediate dimer with varying [Mg^2+^] at 20 mM TRIS pH 7.4 and 150 mM KCl (left). Variation of the fraction of molecules exhibiting the intermediate dimer with varying [K^+^] at 20 mM TRIS pH 7.4, 5 mM Mg^2+^ and 150 mM KCl (right). (**c**) Kinetics of the intermediate dimer formation (*k*_dock_, left) and dissociation (*k*_undock_, left) at varying [RNA].

Our labeling strategy enables us to study the dynamic behavior of the KC as well as its progression toward the extended RNA duplex (ED, Figure 1). With this labeling scheme, ED is expected to result in a high static FRET state, while the KC is expected to yield a mid FRET state. A smFRET time trajectory (Figure 4a, left) shows the progression to the ED conformation (static FRET = 1.0) following multiple associations and dissociations of monomer hairpins to KC. Under standard conditions (5 mM Mg^2+^, 150 mM K^+^, 20 mM TRIS pH 7.5, 22°C), only 13% of trajectories reach the ED state. However, under these conditions, we also observe 32% of trajectories exhibiting dynamic excursions between FRET 1.0 and 0.4 (Figure 4a, right). Control experiments confirm that this behavior is never observed with pre-annealed ED complexes, ruling out possible photophysical artifacts caused by the local environment in the ED conformation. Therefore, this dynamic population is likely to represent a different folded state that brings the fluorophores in close proximity, such as a bent KC (similar to the TAR–TAR* complex (52–54)) or a cruciform intermediate initiated by fraying of the hairpins’ stems (55).

The fraction of trajectories exhibiting this intermediate conformation increases with Mg^2+^ ion concentration, indicating that Mg^2+^ ions stabilize this population (Figure 4b, left). Measuring the intermediate docking and undocking rate constants as a function of RNA concentration (Figure 4c and Supplementary Figure S6) shows that docking and undocking is a unimolecular process with respect to RNA and Mg^2+^.

### Magnesium ions are required to form the bent intermediate

To assess the effect of monovalent and divalent ions on the folding pathway, we determined the fraction of each observed state (KC, intermediate bent conformation and extended) with varying [K^+^] and [Mg^2+^] in the presence of saturating concentrations of free DIS2. The Mg^2+^ titration was performed at 150 mM background K^+^ ions, while the K^+^ titration was performed in a 5 mM background of Mg^2+^ ions. At low [Mg^2+^], ∼75% of molecules adopt the KC conformation, and only 10–20% form the intermediate or the ED conformation. Increasing the [Mg^2+^] to 50 mM (Figure 4b, left panel) shifts the KC population to the intermediate state, making both fractions equally populated (40%). This data suggest that Mg^2+^ ions promote conversion of KC into the bent intermediate. At high monovalent concentrations (>500 mM), the amount of bent intermediate is highly reduced (∼15%), confirming that Mg^2+^ is required for the transition into the intermediate form.

### A stem mutant rules out a possible cruciform intermediate

**Figure 5.**
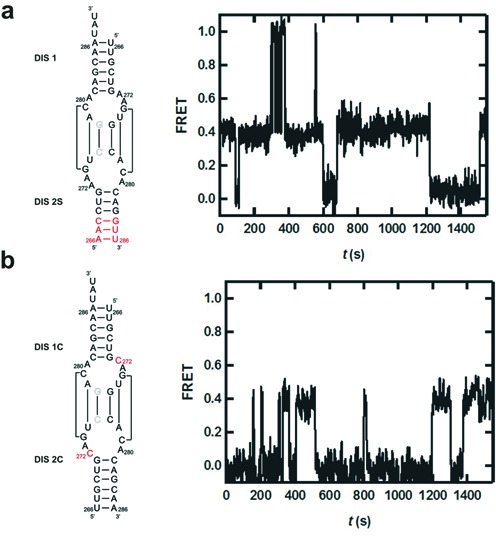
Loop and stem mutant studies to characterize the bent KC. (**a**) A stem mutation (red bases, left) stalls KC dimerization but not the formation of the dynamic bent intermediate, indicating that the bent intermediate does not involve a cruciform conformation. Characteristic time trajectory (right) revealing the formation of the bent KC for the stem mutant. (**b**) A272C mutation (red, left) and characteristic time trajectory (right) reveal the absence of bent intermediate, indicating that the highly conserved flanking purines play an important role in the stabilization of this intermediate.

To characterize the structure of the intermediate and to distinguish between the possible conformations that bring the fluorophores in close proximity, we designed a hairpin mutant where the three terminal base pairs were flipped (Figure 5a), which prevents the formation of the ED through a cruciform (55) or any other intermediate. Experiments with this mutant reveal that 35% of molecules still exhibit dynamic excursions between FRET 1.0 and FRET 0.4. This result clearly rules out the formation of a cruciform intermediate in our experimental conditions and supports the bent KC conformation (Figure 5a). Additional experiments with the mutant also reveal that the excursion times in the FRET 1.0 state are reduced relative to the wild type (Figure 4 and Supplementary Table S1), suggesting that long range interactions between the complementary stem region of the RNA hairpins in KC may contribute to the stability of the bent kissing structure (Figure 5). To further test this model, we introduced both donor and acceptor fluorophores on a single hairpin (DIS-2) and observed the ED formation (Supplementary Figure S7). In this scheme, the model predicts that both the KC and the bent complex are characterized by high FRET values (the stem remains base paired), whereas the ED should yield intermediate FRET. The data support the kinetic model and shows that the stem regions of the hairpin RNA remain base paired until the irreversible formation of the ED.

In summary, these data suggest that the dynamic intermediate transiently adopts a bent KC conformation (FRET 1.0) in equilibrium with the coaxially stacked conformation (FRET 0.4, Figure 1b).

### 3D model of a DIS kissing-loop bent intermediate

**Figure 6.**
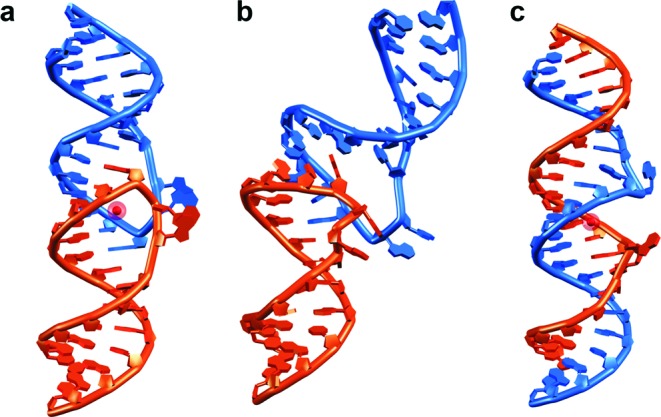
3D architecture of HIV-1 DIS RNA dimers along the isomerization pathway. The two strands are shown in orange and blue. (a) The DIS kissing complex as observed in crystal structures (PDB 1XPF). (b) Molecular model of a bent kissing complex based on a TAR complex (44). (c) Extended duplex as observed in crystal structures (PDB 462D). Hexahydrated magnesium ions observed in X-ray structures are shown as red spheres.

Modeling of a possible bent DIS KC was carried out starting from the kinked TAR–TAR* kissing-loop NMR structure and the coaxially stacked DIS kissing-loop complex crystal structure. Structure modeling showed that only limited accommodations are required to induce a significant bending of the coaxially stacked KC. In particular, flanking adenines 272 and 273 5′ of the self-complementary sequence can maintain an extra-helical conformation in the bent KC similar to the one observed in the coaxial one (Figure 6).

### A272 is essential for stability and bending of the KC

It has been established that the stability of the KC is strongly dependent on the three highly conserved purines flanking the self-complementary sequence (13,14). In particular, it was suggested that A272 has a large impact on the dynamics and local conformational changes of the KC (14,24). We investigated the effect of A272 by mutating this base to a cytidine (A272C, Figure 5b). Under standard buffer conditions, the A272C mutant exhibits no long-lived KC population (Supplementary Table S1), similar to the wild-type hairpins in the absence of Mg^2+^ ions. This result is consistent with the binding of a Mg^2+^ ion in the vicinity of A272, thus stabilizing the flipped out conformation of the base. This, in turn, can stabilize the KC through inter-helical adenine stacking interactions as observed in crystal structures (31).

Furthermore, the introduction of A272C mutation to one of the RNA hairpins significantly decreases (<10%) the population of molecules in the bent intermediate conformation. This asserts the fact that the metastable KC is an obligatory step that is required for the transition into the bent KC.

## DISCUSSION

RNA KCs/loop–loop interactions play a major role in multistep RNA folding pathways. Kinetics and thermodynamics of RNA loop–loop interactions have been previously evaluated using multiple techniques such as surface plasmon resonance (SPR), electrospray ionization mass spectrometry (ESI-MS), ITC, NMR, bulk fluorescence measurements, electrospray ionization-Fourier transform mass spectrometry and UV melting (23,24,48,55–58). These studies concluded that isomerization proceeds without complete disruption of the loop–loop helix. However, details of the exact mechanism remained unclear. Studies using model RNA hairpins have shown that there is a large kinetic barrier for the conversion of KC to the ED (48).

In this study, we have used smFRET to characterize the dimerization mechanism of HIV-1 in real time using the minimal RNA sequence responsible for the viral genome dimerization. Our smFRET kinetic data reveal that the formation and dissociation of HIV-1 KC is an extraordinarily slow process under near-physiological conditions *in vitro*. We observed multiple association and dissociation steps from the monomer to the KC dimer. The dissociation kinetics is highly dependent on salt conditions. In the presence of Mg^2+^, highly stable KC dimers were observed, which agrees with previous reports showing that metastable kissing dimers are formed in the presence of divalent metal ions (23). It has been also shown that, in the presence of Mg^2+^, conversion of kissing dimer to the ED requires nucleocapsid protein (23). Our results clearly show that the conversion of KC to ED can be achieved slowly in the absence of nucleocapsid protein after multiple dissociation and re-association steps. Our results with the A272C mutation shows that, in the absence of A272, kissing dimers are unable to gain high stability even in the presence of high concentrations of [Mg^2+^]. This confirms the essential role played by this universally conserved adenine in the formation of a stable kissing-loop complex (15,24,31). We propose that adenine base flipping and stabilization of flipped adenines upon Mg^2+^ binding might be the cause of the observed high stability of the kissing dimer in the presence of Mg^2+^. In support of this hypothesis, it has been shown that A272 can be protonated and this might promote the loop dynamics and the conversion to ED (24).

**Figure 7.**
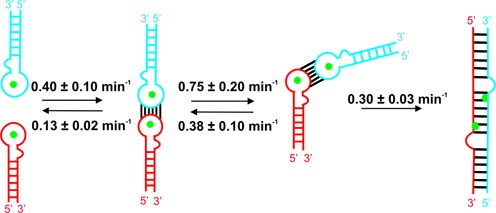
Minimal kinetic model for the HIV-1 RNA dimerization with the observed bent dimer. First the KC forms, followed by the formation of a dynamic bent intermediate, which eventually leads to the formation of a stable extended duplex. Magnesium ions (green spheres) favor the bent KC intermediate formation.

The smFRET trajectories also reveal the presence of an intermediate in the transition from KC to the extended RNA duplex. Experiments performed with inverted DIS stem sequences clearly eliminate the possibility of cruciform intermediates as observed with monomeric DIS hairpin mutants (55). Based on the smFRET data, we propose that the folding intermediate corresponds to a bent KC (Figure 6 and 7), similar to a TAR complex structure (44) or ColEl plasmid specific RNA I and RNA II transcripts (59,60). A similar concept of a bent KC retaining the WC base pairing at the loop interaction and the C2 symmetry of the loop–loop interface that facilitates the duplex formation via KC has been suggested previously based on NMR data (61). A similarly bent KC transition state has also been proposed in the NCp7-chaperoned dimerization pathway (23). Alternatively, the bent intermediate may resemble a structure suggested in a recent NMR study, where the DIS hairpin base pairing remains intact and inter-stem interactions are facilitated as a result of KC bending, which brings the two stems in close proximity (62). In addition, there is *in vivo* evidence for an intermediate HIV-1 gRNA dimer on the path from immature gRNA dimer to mature gRNA dimer inside the HIV-1 particle (63). We built a molecular model of a DIS KC intermediate based on the TAR complex (44) by keeping the angle between both hairpins constant (Figure 6). In this model, A272 and A273 are in a flipped out configuration and stacked onto each other as observed in KC crystal structures, showing the feasibility of bending the DIS KC with perfectly coaxially stacked hairpins thanks to the plasticity provided by unpaired adenines. This is in line with previous studies highlighting the dynamics of these purines within the KC (24,32,64). This molecular model yields an inter-stem distance of ∼49 Å. However, based on the measured FRET efficiency (0.9–1.0), we estimate the distance between the ends of the two hairpin stems to be ≤41 Å (assuming κ^2^ = ⅔). Therefore, we propose that the observed bent KC adopts a conformation in between the model in Figure 6 and the completely bent KC conformation proposed by Dethoff *et al.* (62).

Monovalent and divalent ion titrations clearly show that Mg^2+^ ions are required for the formation of the proposed bent KC. Our data also highlight the important role of the universally conserved adenine 272, 5′ of the loop. Experiments performed with the DIS A272C mutant show that the KC formation is strongly hindered, thus transition to bent KC or the ED cannot be readily achieved even at high Mg^2+^ concentrations.

## CONCLUSIONS

The ability to characterize the folding pathway of HIV-1 RNA *in vitro* is critical to understanding the viral RNA dimerization during viral assembly. We have demonstrated a single-molecule fluorescence resonance energy transfer assay to monitor the dimerization of minimal HIV-1 RNA sequence containing DIS. Our smFRET data revealed that the bimolecular association rate constant of the two hairpin RNAs is 1.3 × 10^5^ M^−1^ s^−1^. This formation rate is independent of the Mg^2+^ and increases linearly with RNA concentration, which confirms that the formation is a diffusion-controlled reaction. The important role of Mg^2+^ for the DIS dimerization has been established by various bulk analysis methods (22,23,50,65). Our study supports the hypothesis that, in solution, magnesium binds the DIS with an equilibrium dissociation constant near 5 mM. Magnesium binding stabilizes the kissing interaction and the KC dissociation rates significantly increase (1.5 ± 0.3 min^−1^) in the absence of magnesium. We observe magnesium bound metastable KC population with extreme slow dissociation rate (0.12 ± 0.02 min^−1^). Our smFRET analysis reveals that HIV-1 RNA dimerization occurs through a three-step folding pathway in which the RNA KC shifts to a bent kissing conformation that leads to the formation of the extended RNA duplex via interaction through stems. We propose a 3D model of a possible bent DIS KC intermediate as expected from smFRET data. Our data also confirm that docking of the KC to form a bent conformation is independent of Mg^2+^ and RNA concentrations, although it requires the presence of Mg^2+^, indicating that bending is a unimolecular process. During maturation of the viral particle, the nucleocapsid protein (NCp7) chaperones the dimerization pathway. Thus, ongoing experiments are currently focused at studying the role of NCp7 in the dimerization pathway at single-molecule level. The mechanistic insights gained from these experiments represent significant progress toward understanding the HIV-1 dimerization mechanism and might help the rational development of new ligands targeting the HIV-1 DIS RNA (66).

## SUPPLEMENTARY DATA

Supplementary Data are available at NAR Online.

SUPPLEMENTARY DATA
