# Supplementary material for: HIV-1 DIS stem loop forms an obligatory bent kissing intermediate in the dimerization pathway
Source: Nucleic Acids Res. 2014 May 9;42(11):7281–9. doi: 10.1093/nar/gku332 (PMC4066764; doi:10.1093/nar/gku332)
Supplement: SUPPLEMENTARY DATA [file supp_42_11_7281__index.html]

SUPPLEMENTARY DATA 

# HIV-1 DIS stem loop forms an obligatory bent kissing intermediate in the dimerization pathway

## SUPPLEMENTARY DATA

**Files in this Data Supplement:**

- Supplementary Data
