# Supplementary material for: HIV-1 DIS stem loop forms an obligatory bent kissing intermediate in the dimerization pathway
Source: Nucleic Acids Res. 2014 May 9;42(11):7281–9. doi: 10.1093/nar/gku332 (PMC4066764; doi:10.1093/nar/gku332)
Supplement: SUPPLEMENTARY DATA [file supp_gku332_nar-03499-f-2013-File009.docx]

**HIV-1 DIS stem loop forms an obligatory bent kissing intermediate in the dimerization pathway**

Hansini Mundigala,^1^ Jonathan B. Michaux,^1^ Andrew L. Feig,^1,^* Eric Ennifar^2,^* and David Rueda^1,3,4,^*

^1^ Department of Chemistry, Wayne State University, Detroit, MI 48236, USA

^2^ Architecture et Réactivité de l’ARN, Université de Strasbourg, Institut de Biologie Moléculaire et Cellulaire du CNRS, F-67084 Strasbourg, France.

^3^ Department of Medicine, Section of Virology, Imperial College, London W12 0NN, UK

^4^ Single Molecule Imaging Group, MRC Clinical Sciences Center, Imperial College, London W12 0NN, UK

* Corresponding author: david.rueda@imperial.ac.uk, e.ennifar@ibmc-cnrs.unistra.fr or afeig@chem.wayne.edu

**Supplementary Table 1.** Effect of base mutations on DIS and the stem on dimerization**^1^**

| **RNA** | | Rate constants^2^ | | | | | **Fraction of**^3^ | | |
| --- | --- | --- | --- | --- | --- | --- | --- | --- | --- |
| **HP1**^4^ | **HP2** | k’_on_ | k_off,1_ | k_off,2_ | k_dock_ | k_undock_ | KC | BKC | ED |
| DIS1 | DIS2 | 0.3 ± 0.1 | 1.0 ± 0.4 | 0.2 ± 0.1 | 0.8 ± 0.2 | 0.4 ± 0.1 | 0.55 | 0.32 | 0.12 |
| DIS1C | DIS2 | 2.1 ± 0.5 | 3.0 ± 0.8 | - | - | - | 0.79 | 0.03 | 0.18 |
| DIS1 | DIS2C | 2.2 ± 0.7 | 3.0 ± 0.4 | - | - | - | 0.73 | 0.06 | 0.20 |
| DIS1C | DIS2C | 1.9 ± 0.4 | 1.3 ± 0.3 | - | - | - | 0.79 | 0.08 | 0.13 |
| DIS1 | DIS2S | 0.8 ± 0.1 | 1.0 ± 0.2 | 0.3 ± 0.1 | 3.5 ± 0.2 | 53 ± 6 | 0.65 | 0.35 | - |

^1^ Experimental conditions are: 20 mM TRIS (pH 7.5), 150 mM KCl, 5 mM Mg^2+^

^2^ Rate constant units are all in min^-1^

^3^ KC (Kissing complex), BKC (Bent kissing complex) and ED (Extended duplex)

^4^ HP1 is immobilized on quartz slide


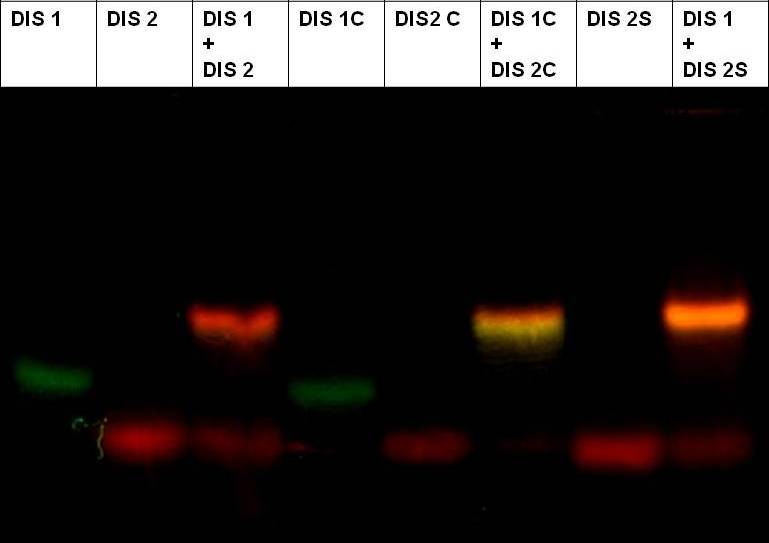


**Supplementary Figure 1**: Native gel analysis of RNA sequences used in this study under near physiological experimental conditions (20 mM TRIS pH 7.4, 5 mM Mg^2+^ and 150 mM KCl) . In the presence of wild type HIV-1 sub type F sequence with modified palindromic sequence, monomer hairpin RNA is observed (Lane 1, Lane 2). When DIS1 and DIS2 RNA are allowed to interact, a mix of kissing complex (KC) and bent kissing complex (bent KC) is observed as a dark orange FRET band in lane 3. Lane 4 and 5, respectively, show the monomer RNA of DIS1C and DIS2C with the A272C mutation. In the presence of both Adenine mutated hairpins (Lane 6), only KC is formed and can be distinguished by prominent yellow FRET band. Lane 7 contains the Cy5 labeled stem mutated RNA DIS2S.


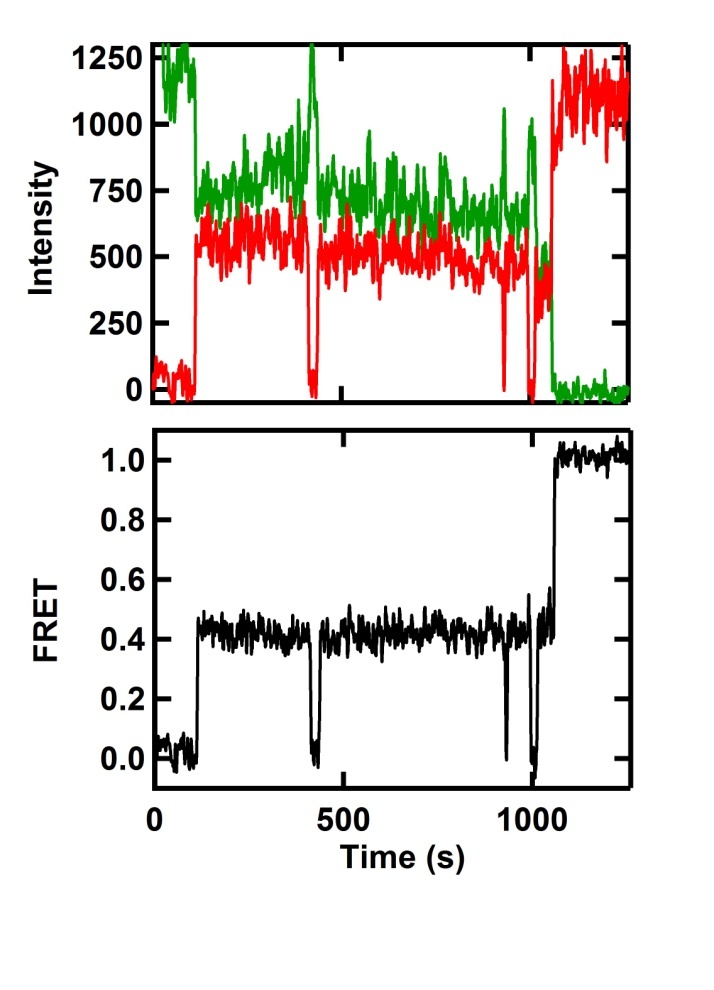


**Supplementary Figure 2**: Variation of donor (green) and acceptor (red) intensities of two RNA hairpins dwelling between kissing complex and monomer forms and ultimately forming extended RNA duplex. FRET trajectory corresponding to the observed intensity variation is also shown.

**
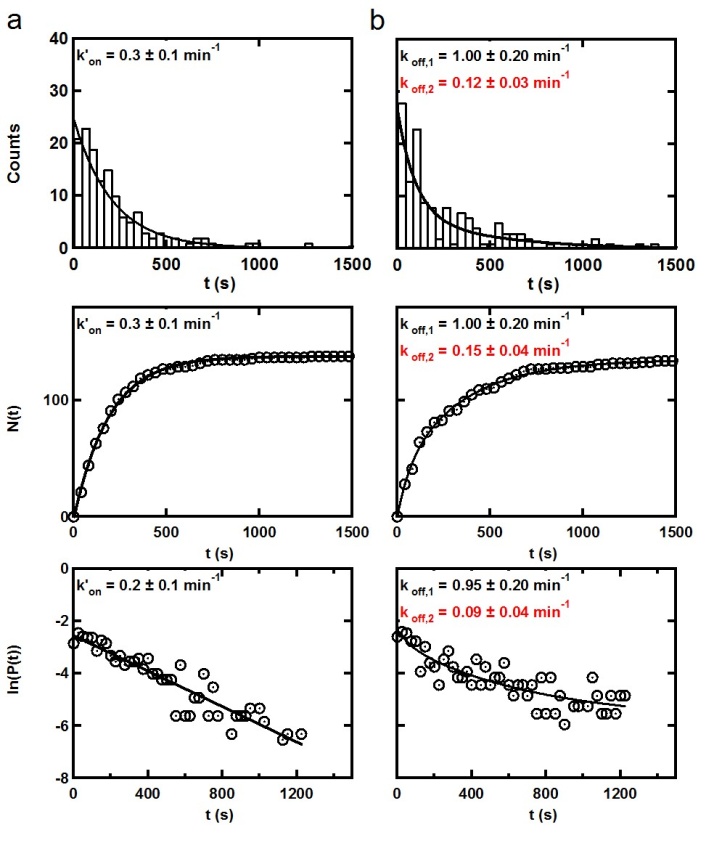
**

**Supplementary Figure 3:** Single-molecule kinetic analysis. Dwell time distributions in the kissing-loop complex (τ_on_) and dissociated state (τ_off_) are fit to single/double exponential decays to obtain the pseudo-fist order binding and dissociation rate constants k_on_ and k_off_ (in 20 mM TRIS pH 7.4, 10 mM Mg^2+^ and 150 mM KCl). Three graphical approaches were used to obtain rate constants: dwell time histograms (top), integrated dwell times (middle) and log-linear analysis of dwell times (bottom).


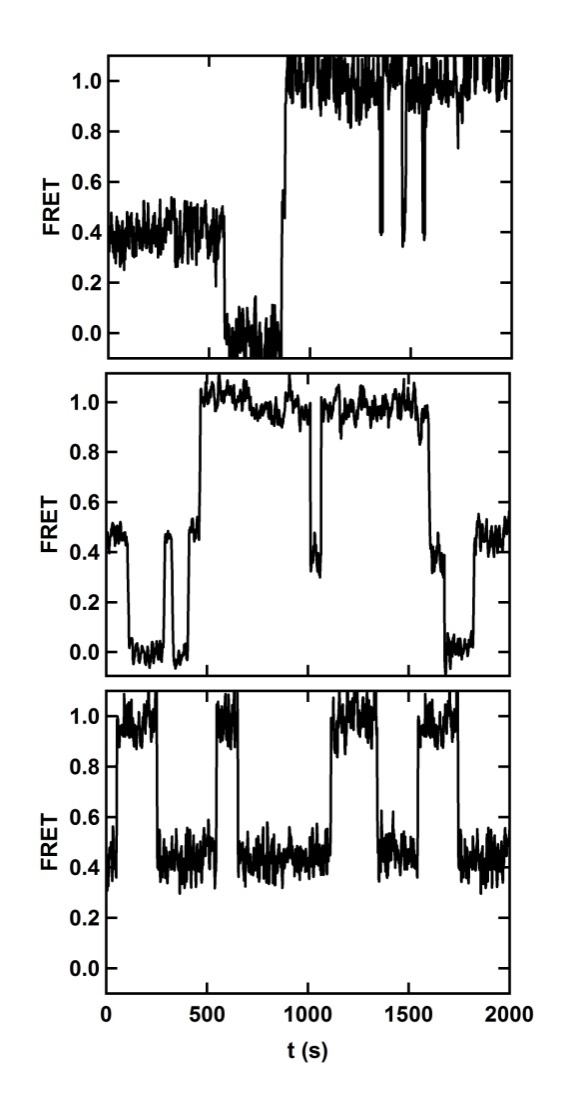


**Supplementary Figure 4**: Types of the dynamic high FRET traces observed during the experiments under near physiological conditions (20 mM TRIS pH 7.4, 5 mM Mg^2+^ and 150 mM KCl). Each of these corresponds to the bent kissing complex intermediate. Top trace corresponds to the conversion of the kissing complex to the extended RNA duplex via a bent kissing intermediate. Trace in the middle shows the formation of the bent kissing intermediate from the kissing complex that comes back to the monomer-kissing complex equilibrium. The bottom trace shows a highly dynamic bent kissing intermediate that dwells between the docked and undocked forms for the duration of the experiment


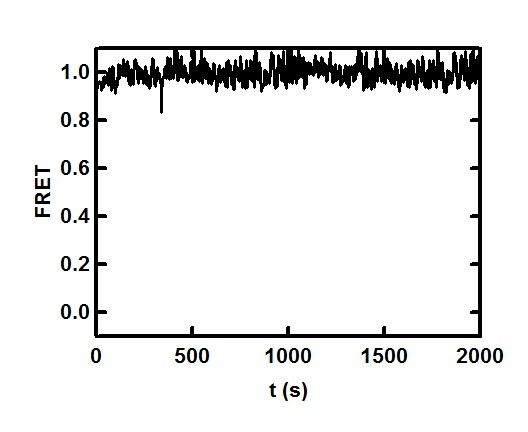


**Supplementary Figure 5**: A Characteristic smFRET trace of a pre-annealed extended RNA duplex. DIS 1 and DIS 2 hairpin RNA were mixed, heated to 94 ̊C, and allowed to cool gradually under near physiological conditions 20 mM TRIS pH 7.4, 5 mM Mg^2+^ and 150 mM KCl. This process allows the two RNA strands to be annealed and favors the formation of extended RNA duplex.


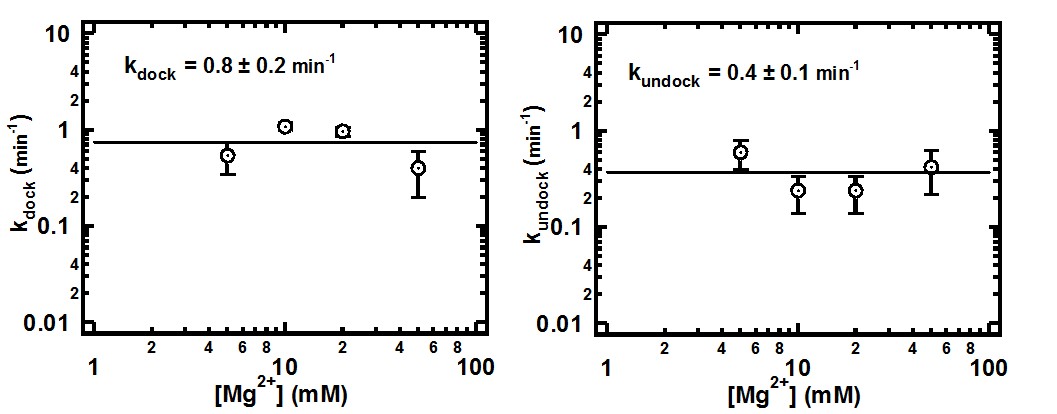


**Supplementary Figure 6**: Kinetics of the intermediate dimer formation and dissociation in 20 mM TRIS pH 7.4, 150 mM KCl, and varying [Mg^2+^]. Docking and undocking rate constants in the dynamic bent intermediate state are independent of [Mg^2+^].





**Supplementary Figure 7:** (a) Schematic diagram of smFRET experiments with both donor and acceptor fluorophores placed on DIS-2. According to the proposed model both the kissing complex and the bent kissing complex should exhibit high FRET (~1.0), whereas the extended duplex formation should result in intermediate FRET (~0.5). (b) Under conditions that promote formation of all species (20 mM TRIS pH 7.4, 5 mM Mg^2+^ and 150 mM KCl, see Figure 3), three trajectories types are observed: static high FRET (top), single transitions from high (~1.0) to intermediate (~0.5) FRET indicating extended duplex formation (middle), and static intermediate FRET indicating pre-formed extended duplex (bottom). The lack of dynamics in these trajectories indicates that the stem of DIS-2 remains stably base paired until the extended duplex is formed. Kinetics of extended duplex formation: FRET histograms reveal the formation extended duplex (0.5 FRET) as a function of time (c). The observed kinetics (d) is consistent with previous measurements and the 60-minute lag period is in agreement with the presence of obligatory intermediates along the pathway.
